# Data Mining in Healthcare: Applying Strategic Intelligence Techniques to Depict 25 Years of Research Development

**DOI:** 10.3390/ijerph18063099

**Published:** 2021-03-17

**Authors:** Maikel Luis Kolling, Leonardo B. Furstenau, Michele Kremer Sott, Bruna Rabaioli, Pedro Henrique Ulmi, Nicola Luigi Bragazzi, Leonel Pablo Carvalho Tedesco

**Affiliations:** 1 Graduate Program of Industrial Systems and Processes, University of Santa Cruz do Sul, Santa Cruz do Sul 96816-501, Brazil; maikel.kolling@gmail.com (M.L.K.); sott.mk@gmail.com (M.K.S.); 2 Department of Industrial Engineering, Federal University of Rio Grande do Sul, Porto Alegre 90035-190, Brazil; leonardofurstenau@mx2.unisc.br; 3 Department of Medicine, University of Santa Cruz do Sul, Santa Cruz do Sul 96816-501, Brazil; brunarabbaioli@gmail.com; 4 Department of Computer Science, University of Santa Cruz do Sul, Santa Cruz do Sul 96816-501, Brazil; pedroh.u@hotmail.com; 5 Laboratory for Industrial and Applied Mathematics (LIAM), Department of Mathematics and Statistics, York University, Toronto, ON M3J 1P3, Canada

**Keywords:** data mining, industry 4.0, healthcare 4.0, bibliometrics, science mapping, strategic intelligence, co-word analysis, SciMAT

## Abstract

In order to identify the strategic topics and the thematic evolution structure of data mining applied to healthcare, in this paper, a bibliometric performance and network analysis (BPNA) was conducted. For this purpose, 6138 articles were sourced from the Web of Science covering the period from 1995 to July 2020 and the SciMAT software was used. Our results present a strategic diagram composed of 19 themes, of which the 8 motor themes (‘NEURAL-NETWORKS’, ‘CANCER’, ‘ELETRONIC-HEALTH-RECORDS’, ‘DIABETES-MELLITUS’, ‘ALZHEIMER’S-DISEASE’, ‘BREAST-CANCER’, ‘DEPRESSION’, and ‘RANDOM-FOREST’) are depicted in a thematic network. An in-depth analysis was carried out in order to find hidden patterns and to provide a general perspective of the field. The thematic network structure is arranged thusly that its subjects are organized into two different areas, (i) practices and techniques related to data mining in healthcare, and (ii) health concepts and disease supported by data mining, embodying, respectively, the hotspots related to the data mining and medical scopes, hence demonstrating the field’s evolution over time. Such results make it possible to form the basis for future research and facilitate decision-making by researchers and practitioners, institutions, and governments interested in data mining in healthcare.

## 1. Introduction

Deriving from Industry 4.0 that pursues the expansion of its autonomy and efficiency through data-driven automatization and artificial intelligence employing cyber-physical spaces, the Healthcare 4.0 portrays the overhaul of medical business models towards a data-driven management [1]. In akin environments, substantial amounts of information associated to organizational processes and patient care are generated. Furthermore, the maturation of state-of-the-art technologies, namely, wearable devices, which are likely to transform the whole industry through more personalized and proactive treatments, will lead to a noteworthy increase in user patient data. Moreover, the forecast for the annual global growth in healthcare data should exceed soon 1.2 exabytes a year [1]. Despite the massive and growing volume of health and patient care information [2], it is still, to a great extent, underused [3].

Data mining, a subfield of artificial intelligence that makes use of vast amounts of data in order to allow significant information to be extracted through previously unknown patterns, has been progressively applied in healthcare to assist clinical diagnoses and disease predictions [2]. This information has been known to be rather complex and difficult to analyze. Furthermore, data mining concepts can also perform the analysis and classification of colossal bulks of information, grouping variables with similar behaviors, foreseeing future events, amid other advantages for monitoring and managing health systems ceaselessly seeking to look after the patients’ privacy [4]. The knowledge resulting from the application of the aforesaid methods may potentially improve resource management and patient care systems, assist in infection control and risk stratification [5]. Several studies in healthcare have explored data mining techniques to predict incidence [6] and characteristics of patients in pandemic scenarios [7], identification of depressive symptoms [8], prediction of diabetes [9], cancer [10], scenarios in emergency departments [11], amidst others. Thus, the utilization of data mining in health organizations ameliorates the efficiency of service provision [12], quality of decision making, and reduces human subjectivity and errors [13].

The understanding of data mining in the healthcare sector is, in this context, vital and some researchers have executed bibliometric analyses in the field with the intention of investigating the challenges, limitations, novel opportunities, and trends [14,15,16,17]. However, at the time of this study, there were no published works that provided a complete analysis of the field using a bibliometric performance and network analysis (BPNA) (see Table 1. In the light of this, we have defined three research questions:

RQ1: What are the strategic themes of data mining in healthcare?
RQ2: How is the thematic evolution structure of data mining in healthcare?
RQ3: What are the trends and opportunities of data mining in healthcare for academics and practitioners?

Thus, with the objective to lay out a superior understanding of the data mining usage in the healthcare sector and to answer the defined research questions, we have performed a bibliometric performance and network analysis (BPNA) to set fourth an overview of the area. We used the Science Mapping Analysis Software Tool (SciMAT), a software developed by Cobo et al. [18] with the purpose of identifying strategic themes and the thematic evolution structure of a given field, which can be used as a strategic intelligence tool. The strategic intelligence, an approach that can enhance decision-making in terms of science and technology trends [19,20,21,22,23,24,25,26,27], can help researchers and practitioners to understand the area and devise new ideas for future works as well as to identify the trends and opportunities of data mining in healthcare.

This research is structured as follows: Section 2 highlights the methodology and the dataset. Section 3 presents the bibliometric performance of data mining in healthcare. In Section 4, the strategic diagram presents the most relevant themes according to our bibliometric indicators as well as the thematic network structure of the motor themes and the thematic evolution structure, which provide a complete overview of data mining over time. Section 5 presents the conclusions, limitations, and suggestions for future works.

## 2. Methodology and Dataset

Attracting attention from companies, universities, and scientific journals, bibliometric analysis enhances decision-making by providing a reliable method to collect information from databases, to transform the aforementioned data into knowledge, and to stimulate wisdom development. Furthermore, the techniques of bibliometric analysis can provide higher and different perspectives of scientific production by using advanced measurement tools and methods to depict how authors, works, journals and institutions are advancing in a specific field of research through the hidden patterns that are embedded in large datasets.

The existing works on bibliometric analysis of data mining in health care in the Web of Science are shown in Table 1, where it is depicted that only three studies have been performed and the differences between these approaches and this work are explained.

### 2.1. Methodology

For this study we have applied BPNA, a method that combines science mapping with performance analysis, to the field of data mining in healthcare with the support of the SciMAT software. This methodology has been chosen in view of the fact that such a combination, in addition to assisting decision-making for academics and practitioners, allows us to perform a deep investigation into the field of research by giving a new perspective of its intricacies. The BPNA conducted in this paper was composed of four steps outlined below.

#### 2.1.1. Discovery of Research Themes

The themes were identified using a frequency and network reduction of keywords. In this process, the keywords were firstly normalized using the Salton’s Cosine, a correlation coefficient, and then clustered through the simple center algorithm. Finally, the thematic evolution structure co-word network was normalized using the equivalence index.

#### 2.1.2. Depicting Research Themes

**Figure 1:**
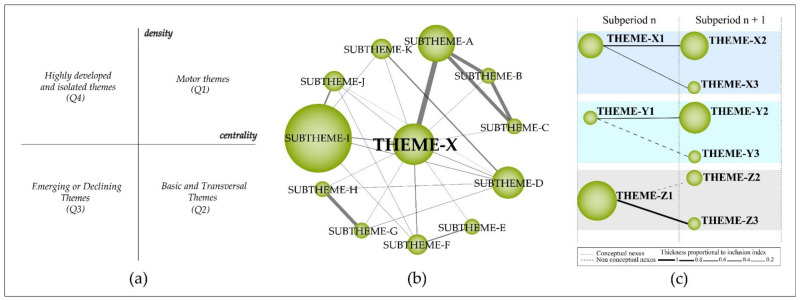
Strategic diagram (**a**). Thematic network structure (**b**). Thematic evolution structure (**c**).

The previously identified themes were then plotted on a bi-dimensional diagram composed of four quadrants, in which the “vertical axis” characterizes the density (D) and the “horizontal axis” characterizes the centrality (C) of the theme [28,29] (Figure 1a) [18,20,25,30,31,32,33].

(a) First quadrant—motor themes: trending themes for the field of research with high development.
(b) Second quadrant—basic and transversal themes: themes that are inclined to become motor themes in the future due to their high centrality.
(c) Third quadrant—emerging or declining themes: themes that require a qualitative analysis to define whether they are emerging or declining.
(d) Fourth quadrant—highly developed and isolated themes: themes that are no longer trending due to a new concept or technology.

#### 2.1.3. Thematic Network Structure and Detection of Thematic Areas

The results were organized and structured in (a) a strategic diagram (b) a thematic network structure of motor themes, and (c) a thematic evolution structure. The thematic network structure (Figure 1b) represents the co-occurrence between the research themes and underlines the number of relationships (C) and internal strength among them (D). The thematic evolution structure (Figure 1c) provides a proper picture of how the themes preserve a conceptual nexus throughout the following sub-periods [23,34]. The size of the clusters is proportional to the number of core documents and the links indicate co-occurrence among the clusters. Solid lines indicate that clusters share the main theme, and dashed lines represent the shared cluster elements that are not the name of the themes [35]. The thickness of the lines is proportional to the inclusion index, which indicates that the themes have elements in common [35]. Furthermore, in the thematic network structure the themes were then manually classified between data mining techniques and medical research concepts.

#### 2.1.4. Performance Analysis

The scientific contribution was measured by analyzing the most important research themes and thematic areas using the h-index, sum of citations, core documents centrality, density, and nexus among themes. The results can be used as a strategic intelligence approach to identify the most relevant topics in the research field.

### 2.2. Dataset

Composed of 6138 non-duplicated articles and reviews in English language, the dataset used in this work was sourced from the Web of Science (WoS) database utilizing the following query string (“data mining” and (“health*” OR “clinic*” OR “medic* OR “disease”)). The documents were then processed and had their keywords, both the author’s and the index controlled and uncontrolled terms, extracted and grouped in accordance with their meaning. In order to remove duplicates and terms which had less than two occurrences in the documents, a preprocessing step was applied to the authors, years, publication dates, and keywords. For instance, the preprocessing has reduced the total number of keywords from 21,838 to 5310, thus improving the bibliometric analysis clarity. With the exception of the strategic diagram that was plotted utilizing a single period (1995–July 2020), in this study, the timeline was divided into three sub-periods: 1995–2003, 2004–2012, and 2013–July 2020.

**Figure 2:**
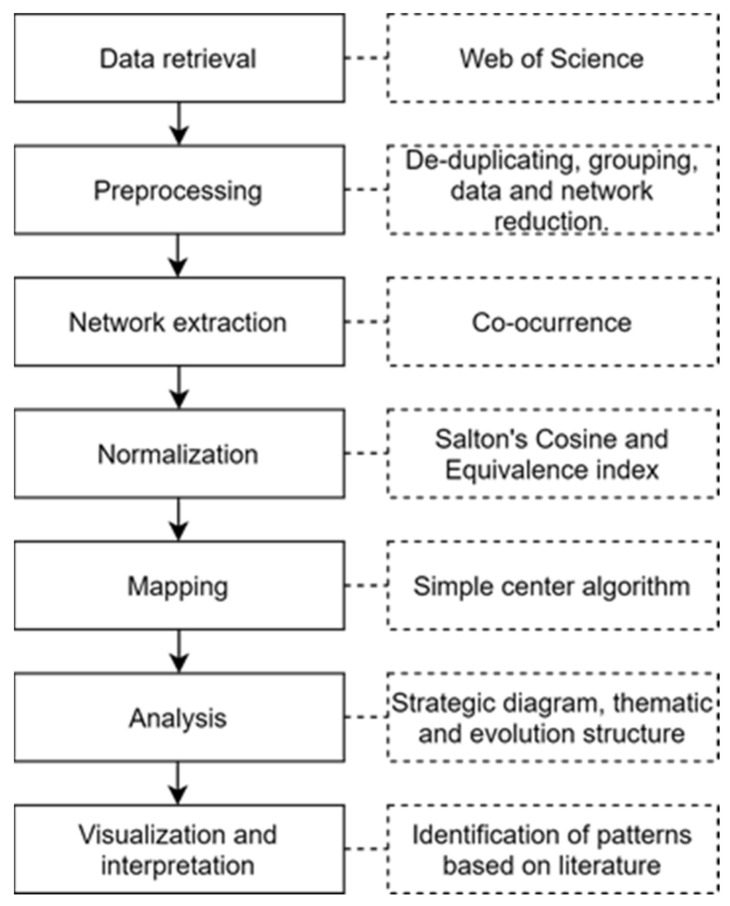
Workflow of the bibliometric performance and network analysis (BPNA).

Subsequently, a network reduction was applied in order to exclude irrelevant words and co-occurrences. For the network extraction we wanted to identify co-occurrence among words. For the mapping process, we used a simple center algorithm. Finally, a core mapper was used, and the h-index and sum citations were selected. Figure 2 shows a good representation of the steps of the BPNA.

## 3. Bibliometric Performance of Data Mining in Healthcare

In this section, we measured the performance of the field of data mining in healthcare in terms of publications and citations over time, the most productive and cited researchers, as well as productivity of scientific journals, institutions, countries, and most important research areas in the WoS. To do this, we used indicators such as: number of publications, sum of citations by year, journal impact factor (JIF), geographic distribution of publications, and research field. For this, we examined the complete period (1995 to July 2020).

### 3.1. Publications and Citations Overtime

**Figure 3:**
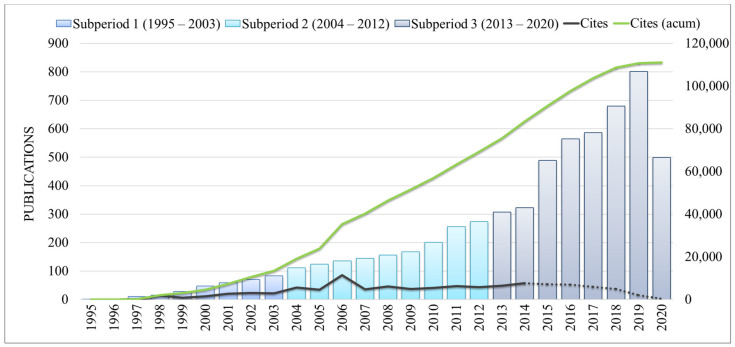
Number of publications over time (1995–July 2020).

Figure 3 shows the performance analysis of publications and citations of data mining in healthcare over time from 1995 to July 2020 in the WoS. The first sub-period (1995–2003) shows the beginning of the research field with 316 documents and a total of 13,483 citations. Besides, the first article in the WoS was published by Szolovits (1995) [36] who presented a tutorial for handling uncertainty in healthcare and highlighted the importance to develop data mining techniques in order to assist the healthcare sector. This sub-period shows a slightly increasing number of citations until 2003 and the year with the highest number of citations was 2002.

The slightly increasing number continues from the first sub-period to the second subperiod (2004–2013) with a total of 1572 publications and 55,734 citations. The year 2006 presents the highest number of citations mainly due to the study of Fawcett [37] which attracted 7762 citations. The author introduced the concept of Receiver Operating Characteristics (ROC). This technique is widely used in data mining to assist medical decision-making.

From the second to the third sub-period, it is possible to observe a huge increase in the number of publications (4250 publications) and 41,821 citations. This elevated increase may have occurred due to the creation of strategies to implement emerging technologies in the healthcare sector in order to move forward with the third digital revolution in healthcare, the so-called Healthcare 4.0 [1,38]. Furthermore, although the citations are showing a positive trend, it is still possible to observe a downward trend from 2014 to 2020. This may happen, as Wang [39] highlights, due to the fact that a scientific document needs three to seven years to reach its peak point of citation [34]. Therefore, this is not a real trend.

### 3.2. Most Productive and Cited Authors

Table 2 displays the most productive and cited authors from 1995 to July 2020 of data mining in healthcare in the WoS. Leading as the most productive researcher in the field of data mining in healthcare is Li, Chien-Feng, a pathologist at Chi Mei Hospital which is sixth-ranked in publication numbers. He dedicates his studies to the molecular diagnosis of cancer with innovative technologies. In the sequence, Acharya, U. Rajendra, ranked in the top 1% of highly cited researchers in five consecutive years (2016, 2017, 2018, 2019, and 2020) in computer science according to Thomson’s essential science indicators, shares second place with Chung, Kyungyong from the Division of Engineering and Computer Science at the Kyonggi University in Su-won-si, South Korea. On the other hand, Bate, Andrew C., a member of the Food and Drug Administration (FDA) Science Council of Pharmacovigilance Subcommittee, which is the fourth-ranked institution in publication count as the most cited researcher with 945 citations. Subsequently, Lindquist, Marie, who monitors global pharmacovigilance and data management development at the World Health Organization (WHO), is ranked second with 943 citations. Last but not least, Edwards, E.R., an orthopedic surgeon at the Royal Australasian College of Surgeons is ranked third with 888 citations. Notably, this study does not demonstrate a direct correlation between the number of publications and the number of citations.

### 3.3. Productivity of Scientific Journals, Universities, Countries and Most Important Research Fields

Table 3 shows the journals that publish studies related to data mining in healthcare. PLOS One is the first ranked with 124 publications, followed by Expert Systems with Applications with 105, and Artificial Intelligence in Medicine with 75. On the other hand, the journal Expert Systems with Applications is the journal that had the highest Journal Impact Factor (JIF) from 2019–2020.

Table 4 shows the most productive institutions and the most productive countries. The first ranked is Columbia University followed by U.S. FDA Registration and Harvard University. In terms of country productivity, United States is the first in the rank, followed by China and England. In comparison with Table 2, it is possible to notice that the most productive author is not related to the most productive institutions (Columbia University and U.S. FDA Registration). Besides, the institution with the highest number of publications is in the United States, which is found to be the most productive country.

Regarding Columbia University, it is possible to verify its prominence in data mining in healthcare through its advanced data science programs, which are one of the best evaluated and advanced in the world. We highlight the Columbia Data Science Society, an interdisciplinary society that promotes data science at Columbia University and the New York City community.

The U.S. FDA Registration has a data mining council to promote the prioritization and governance of data mining initiatives within the Center for Biological Research and Evaluation to assess spontaneous reports of adverse events after the administration of regulated medical products. In addition, they created an Advanced and Standards-Based Network Analyzer for Clinical Assessment and Evaluation (PANACEA), which supports the application of standards recognition and network analysis for reporting these adverse events. It is noteworthy that the FDA Adverse Events Reporting System (FAERS) database is the main resource that identifies adverse reactions in medications marketed in the United States. A text mining system based on EHR that retrieves important clinical and temporal information is also highlighted along with support for the Cancer Prevention and Control Division at the Centers for Disease Control and Prevention in a big data project.

The Harvard University offers online data mining courses and has a Center for Healthcare Data Analytics created by the need to analyze data in large public or private data sets. Harvard research includes funding and providing healthcare, quality of care, studies on special and disadvantaged populations, and access to care.

Table 5 presents the most important WoS subject research fields of data mining in healthcare from 1995 to July 2020. Computer Science Artificial Intelligence is the first ranked with 768 documents, followed by Medical Informatics with 744 documents, and Computer Science Information Systems with 722 documents.

## 4. Science Mapping Analysis of Data Mining in Healthcare

In this section the science mapping analysis of data mining in healthcare is depicted. The strategic diagram shows the most relevant themes in terms of centrality and density. The thematic network structure uncovers the relationship (co-occurrence) between themes and hidden patterns. Lastly, the thematic evolution structure underlines the most important themes of each sub-period and shows how the field of study is evolving over time.

### 4.1. Strategic Diagram Analysis

**Figure 4:**
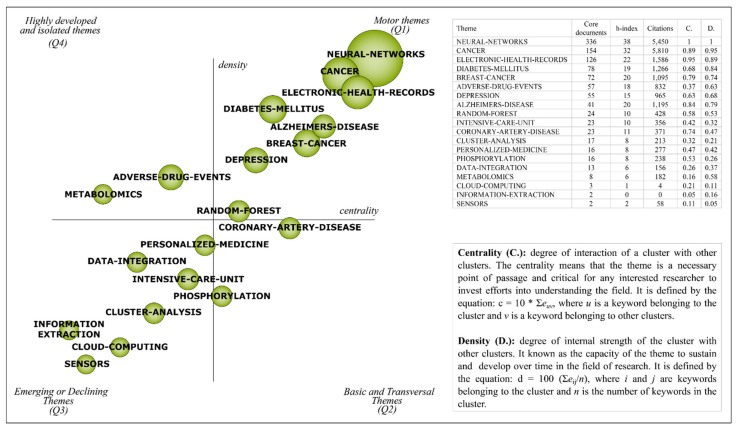
Strategic diagram of data mining in healthcare (1995–July 2020).

Figure 4 presents 19 clusters, 8 of which are categorized as motor themes (‘NEURAL-NETWORKS’, ‘CANCER’, ‘ELETRONIC-HEALTH-RECORDS’, ‘DIABETES-MELLITUS’, ‘ADVERSE-DRUG-EVENTS’, ‘BREAST-CANCER’, ‘DEPRESSION’ and ‘RANDOM-FOREST’), 2 as basic and transversal themes (‘CORONARY-ARTERY-DISEASE’ and ‘PHOSPHORYLATION’), 7 as emerging or declining themes (‘PERSONALIZED-MEDICINE’, ‘DATA-INTEGRATION’, ‘INTENSIVE-CARE-UNIT’, ‘CLUSTER-ANALYSIS’, ‘INFORMATION-EXTRACTION’, ‘CLOUD-COMPUTING’ and ‘SENSORS’), and 2 as highly developed and isolated themes (‘ALZHEIMERS-DISEASE’, and ‘METABOLOMICS’).

Each cluster of themes was measured in terms of core documents, h-index, citations, centrality, and density. The cluster ‘NEURAL-NETWORKS’ has the highest number of core documents (336) and is ranked first in terms of centrality and density. On the other hand, the cluster ‘CANCER’ is the most widely cited with 5810 citations.

### 4.2. Thematic Network Structure Analysis of Motor Themes

**Figure 5:**
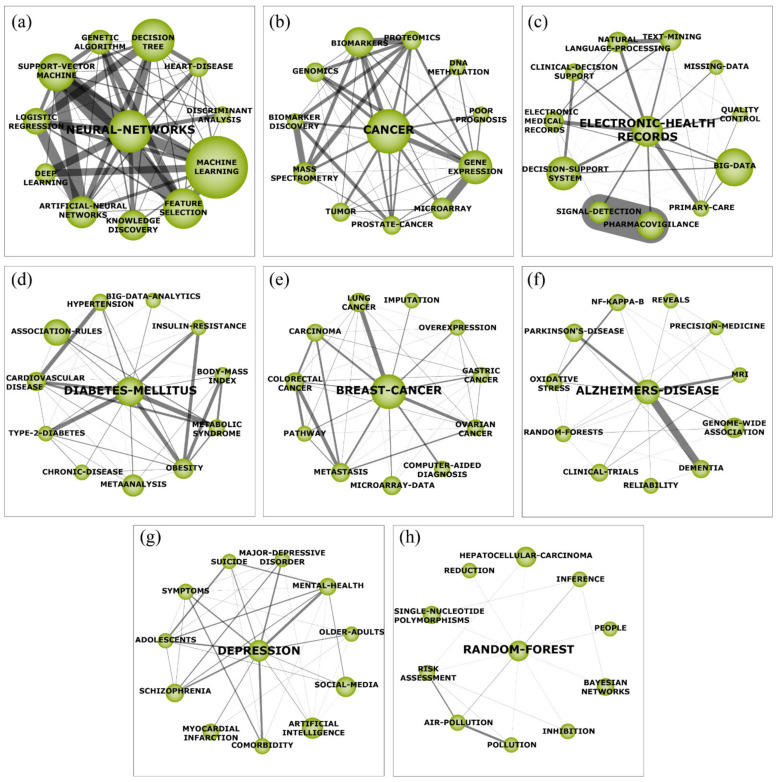
Thematic network structure of mining in healthcare (1995–July 2020). (**a**) The cluster ‘NEURAL-NETWORKS’. (**b**) The cluster ‘CANCER’. (**c**) The cluster ‘ELECTRONIC-HEALTH-RECORDS’. (**d**) The cluster ‘DIABETES-MELLITUS’. (**e**) The cluster ‘BREAST-CANCER’. (**f**) The cluster ‘ALZHEIMER’S DISEASE’. (**g**) The cluster ‘DEPRESSION’. (**h**) The cluster ‘RANDOM-FOREST’.

The motor themes have an important role regarding the shape and future of the research field because they correspond to the key topics to everyone interested in the subject. Therefore, they can be considered as strategic themes in order to develop the field of data mining in healthcare. The eight motor themes are discussed below, and they are displayed below in Figure 5 together with the network structure of each theme.

#### 4.2.1. Neural Network (a)

The cluster ‘NEURAL-NETWORKS’ (Figure 5a) is the first ranked in terms of core documents, h-index, centrality, and density. The ‘NEURAL-NETWORKS’ cluster is strongly influenced by subthemes related to data science algorithms, such as ‘SUPPORT-VECTOR-MACHINE’, ‘DECISION-TREE’, among others. This network represents the use of data mining techniques to detect patterns and find important information correlated to patient health and medical diagnosis. A reasonable explanation for this network might be related to the high number of studies which conducted benchmarking of neural networks with other techniques to evaluate performance (e.g., resource usage, efficiency, accuracy, scalability, etc.) [40,41,42]. Besides, the significant size of the cluster ‘MACHINE-LEARNING’ is expected since neural networks is a type of machine learning. On the other hand, the subtheme ‘HEART-DISEASE’ stands out as the single disease in this network, which can be justified by the high number of researches with the goal to apply data mining to support decision-making in heart disease treatment and diagnosis.

#### 4.2.2. Cancer (b)

The cluster ‘CANCER’ (Figure 5b) is the second ranked in terms of core documents, h-index, and density. On the other hand, it is the first in terms of citations (5810). This cluster is highly influenced by the subthemes related to the studies of cancer genes mutations, such as ‘BIOMAKERS’, ‘GENE-EXPRESSION’, among others. The use of data mining techniques has been attracting attention and efforts from academics in order to help solve problems in the field of oncology. Cancer is known as the disease that kills the most people in the 21st century due to various environmental pollutions, food pesticides and additives [14], eating habits, mental health, among others. Thus, controlling any form of cancer is a global strategy and can be enhanced by applying data mining techniques. Furthermore, the subtheme ‘PROSTATE-CANCER’ highlights that the most efforts of data mining applications focused on prostate cancer’s studies. Prostate cancer is the most common cancer in men. Although the benefits of traditional clinical exams for screening (digital rectal examination, the prostate-specific antigen and blood test and transrectal ultrasound), there is still a lack in terms of efficacy to reduce mortality with the use of such tests [43]. In this sense, data mining may be a suitable solution since it has been used in bioinformatics analyses to understand prostate cancer mutation [44,45] and uncover useful information that can be used for diagnoses and future prognostic tests which enhance both patients and clinical decision-making [46].

#### 4.2.3. Electronic Health Records (HER—c)

The cluster ‘ELECTRONIC-HEALTH-RECORDS’ (Figure 5c) represents the concept in which patient’s health data are stored. Such data are continuously increasing over time, thereby creating a large amount of data (big data) which has been used as input (EHR) for healthcare decision support systems to enhance clinical decision-making. The clusters ‘NATURAL-LANGUAGE-PROCESSING’ and ‘TEXT MINING’ highlight that these mining techniques are the most frequently used with data mining in healthcare. Another pattern that must be highlighted is the considerable density among the clusters ‘SIGNAL-DETECTION’ and ‘PHARMACOVIGILANCE’ which represents the use of data mining to depict a broad range of adverse drug effects and to identify signals almost in real-time by using EHR [47,48]. Besides, the cluster ‘MISSING-DATA’ is related to studies focused on the challenge regarding to incomplete EHR and missing data in healthcare centers, which compromise the performance of several prediction models [49]. In this sense, techniques to handle missing data have been under improvement in order to move forward with the accurate prediction based on medical data mining applications [50].

#### 4.2.4. Diabetes Mellitus (DM—d)

Nowadays, DM is one of the most frequent endocrine disorders [51] and affected more than 450 million people worldwide in 2017 and is expected to grow to 693 million by the year 2045. The same applies for the 850 billion dollars spent just in 2017 by the health sector [52]. The cluster ‘DIABETES-MELLITUS’ (Figure 5d) has a strong association with the risk factor subtheme group (e.g., ‘INSULIN-RESISTENCE’, ‘OBESITY’, ‘BODY-MASS-INDEX’, ‘CARDIOVASCULAR-DISEASE’, and ‘HYPERTENSION’). However, the obesity (cluster ‘OBESITY’) is the major risk factor related to DM, particularly in Type 2 Diabetes (T2D) [51]. T2D shows a prevalence of 90% of worldwide diabetic patients when compared with T1D and T3D, mainly characterized by insulin resistance [51]. Thus, this might justify the presence of the clusters ‘TYPE-2-DIABETES’ and ‘INSULIN-RESISTANCE’ which seems to be highly developed by data mining academics and practitioners. The massive number of researches into all facets of DM has led to the formation of huge volumes of EHR, in which the mostly applied data mining technique is the association rules technique. It is used to identify associations among risk factors [51], thusly justifying the appearance of the cluster ‘ASSOCIATION-RULES’.

#### 4.2.5. Breast Cancer (e)

The cluster ‘BREAST-CANCER’ (Figure 5e) presents the most prevalent type of cancer affecting approximately 12.5% of women worldwide [53,54]. The cluster ‘OVEREXPRESSION’ and ‘METASTASIS’ highlights the high number of studies using data mining to understand the association of overexpression of molecules (e.g., MUC1 [54], TRIM29 [55], FKBP4 [56], etc.) with breast cancer metastasis. Such overexpression of molecules also appears in other forms of cancers, justifying the group of subthemes: ‘LUNG CANCER’, ‘GASTRIC-CANCER’, ‘OVARIAN-CANCER’, and ‘COLORECTALCANCER’. Moreover, the cluster ‘IMPUTATION’ highlight efforts to develop imputation techniques (data missingness) for breast cancer record analysis [57,58]. Besides, the application of data mining to depict breast cancer characteristics and their causes and effects has been highly supported by ‘MICROARRAY-DATA’ [59,60], ‘PATHWAY’ [61], and ‘COMPUTER-AIDED-DIAGNOSIS’ [62].

#### 4.2.6. Alzheimer’s Disease (AD—f)

The cluster ‘ALZHEIMER’S DISEASE’ (Figure 5f) is highly influenced by subthemes related to diseases, such as ‘DEMENTIA’ and ‘PARKINSON’S-DISEASE’. This co-occurrence happens because the AD is a neurodegenerative illness which leads to dementia and Parkinson’s disease. Studies show that the money spent on AD in 2015 was about $828 billion [63]. In this sense, data mining has been widely used with ‘GENOME-WIDE-ASSOCIATION’ techniques in order to identify genes related to the AD [64,65] and prediction of AD by using data mining in ‘MRI’ Brain images [66,67]. The cluster ‘NF-KAPPA-B’ highlights the efforts to identify associations of NF-κB (factor nuclear kappa B) with AD by using data mining techniques which can be used to advance anti-drug developments [68].

#### 4.2.7. Depression (g)

The cluster ‘DEPRESSION’ (Figure 5g) presents a common disease which affects over 260 million people. In the worst case, it can lead to suicide which is the second leading cause of death in young adults. The cluster ‘DEPRESSION’ is a highly associated cluster. Its connections mostly represent the subthemes that have been the research focus of data mining applications [69]. The connection between both the sub theme ‘SOCIAL-MEDIA’ and ‘ADOLESCENTS’, especially in times of social isolation, are extremely relevant to help identify early symptoms and tendencies among the population [70]. Furthermore, the presence of the ‘COMORBIDITY’ and ‘SYMPTONS’ is not surprising given knowledge discovery properties of the data mining field could provide significant insights into the etiology of depression [71].

#### 4.2.8. Random Forest (h)

An ensemble learning method that is used in this study is the last cluster approach, which, among other things, is used for classification. The presence of the ‘BAYESIAN-NETWORK’ subtheme, supported by the connection between both and the ‘INFERENCE’, might represent another alternative to which the applications in data mining using random forest are benchmarked against [72]. Since the ‘RANDOM-FOREST’ (Figure 5h) cluster has barely passed the threshold from a basic and transversal theme to a motor theme, the works developed under this cluster are not yet as interconnected as the previous one. Thus, the theme with the most representativeness is the ‘AIR-POLLUTION’ in conjunction with ‘POLLUTION’, where studies have been performed in order to obtain ‘RISK-ASSESSMENT’ through the exploration of the knowledge hidden in large databases [73].

### 4.3. Thematic Evolution Structure Analysis

**Figure 6:**
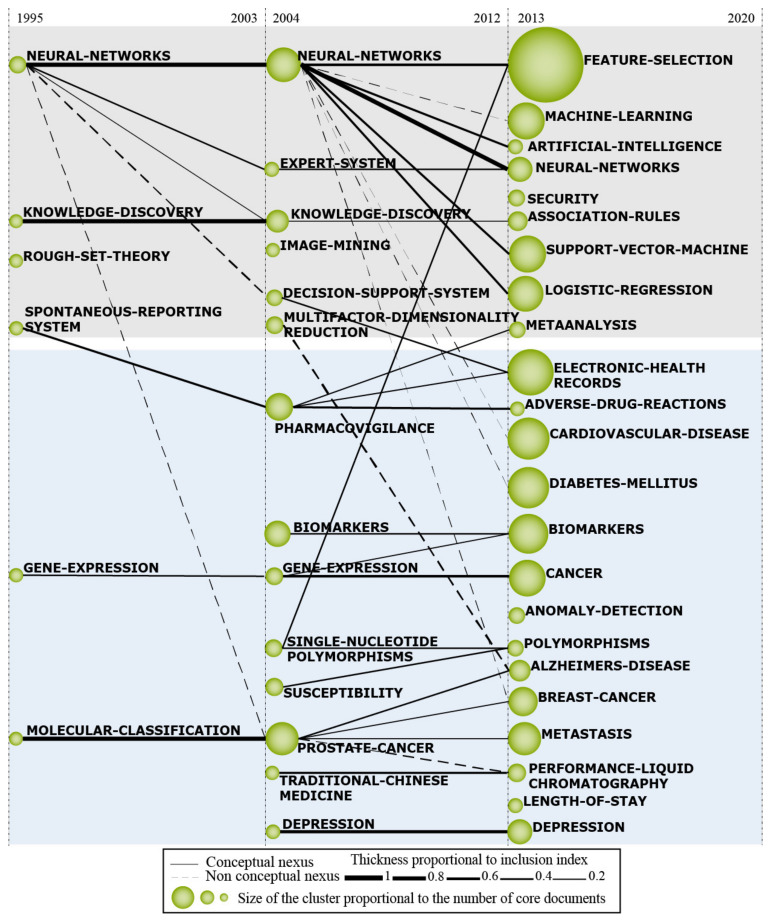
Thematic evolution structure of mining in healthcare (1995–July 2020).

The Computer Science’s themes related to data mining and the medical research concepts, depicted, respectively, in the grey and blue areas of the thematic evolution diagram (Figure 6), demonstrates the evolution of the research field over the different sub-periods addressed in this study. In this way, each individual theme relevance is illustrated through its cluster size as well as with its relationships throughout the different sub-periods. Thus, in this section, an analysis of the different trends on themes will be presented to give a brief insight into the factors that might have influenced its evolution. Furthermore, the proceeding analysis will be split into two thematic areas where, firstly, the grey area (practices and techniques related to data mining in healthcare) will be discussed followed by the blue one (health concepts and disease supported by data mining).

#### 4.3.1. Practices and Techniques Related to Data Mining in Healthcare

The cluster ‘KNOWLEDGE-DISCOVERY’ (Figure 6, 1995–2012), often known as a synonym for data mining, provides a broader view of the field differing in this way from the algorithm focused theme, that is data mining, where its appearance and, later in the third period, its fading could provide a first insight into the overall evolution of the data mining papers applied to healthcare. The occurrence of the cluster knowledge discovery in the first two periods could demonstrate the focus of the application of the data mining techniques in order to classify and predict conditions in the medical field. This gives rise to a competition with early machine learning techniques that could be potentially evidenced through the presence of the cluster ‘NEURAL-NETWORK’, which the data mining techniques could probably be benchmarked against. The introduction of the ‘FEATURE-SELECTION’, ‘ARTIFICIAL-INTELLIGENCE’, and ‘MACHINE-LEARNING’ clusters together with the fading of ‘KNOWLEDGE-DISCOVERY’ could imply the occurrence of a disruption of the field in the third sub-period that has led to a change in the perspective on the studies.

One instance that could represent such a disruption could have been a well-known paper published by Alex Krizhevsky, Ilya Sutskever, and Geoffrey Hinton [74], where a novel technique in neural networks was firstly applied to a major image recognition competition. A vast advantage over the other algorithms that have been used was obtained. The connection between the work previously mentioned and its impact on the data mining on healthcare research could be majorly supported by the disappearance of the cluster ‘IMAGE- MINING’ of the second sub-period which has no connections further on. Furthermore, the presence of the clusters ‘MACHINE-LEARNING’, ‘ARTIFICIAL-INTELLIGENCE’, ‘SUPPORT-VECTOR-MACHINES’, and ‘LOGISTIC-REGRESSION’ may be the evidence of a shift of focus on the data mining community for health care where, besides attempting to compete with machine learning algorithms, they are now striving to further improve the results previously obtained with machine learning through data mining. Moreover, given the presence of the colossal feature selection cluster, which circumscribes algorithms that enhance classification accuracy through a better selection of parameters, this trend could be given credence in consequence of its presence since it may be encompassing publications from the formerly stated clusters.

Although still small, the presence of the cluster ‘SECURITY’ in the last sub-period (Figure 6, 2013–2020) is, at the very least, relevant given the sensitive data that is handled in the medical space, such as patient’s history and diseases. Above all, the recent leaks of personal information have devised an ever-increasing attention to this topic focusing on, among other things, the de-identification of the personal information [75,76,77]. These kind of security processes allow, among others, data mining researchers to make use of the vast sensitive information that is stored in hospitals without any linkage that could associate a person to the data. For instance, the MIMIC Critical Care Database [78], an example of a de-identified database, has been allowing further research into many diseases and conditions in a secure way that would otherwise have been extremely impaired due to data limitations.

#### 4.3.2. Health Concepts and Disease Supported by Data Mining

The cluster ‘GENE-EXPRESSION’ stands out in the first period and second period (Figure 6, 1995–2012) of medical research concepts and establishes strong co-occurrence with the cluster ‘CANCER’ in the third sub-period. This link can be explained by research involving the microarray technology, which makes it possible to detect deletions and duplications in the human genome by analyzing the expression of thousands of genes in different tissues. It is also possible to confirm the importance of genetic screening not only for cancer, but for several diseases, such as ‘ALZHEIMER’ and other brain disorders, thereby assisting in preventive medicine and enabling more efficient treatment plans [79]. For example, a research was carried out to analyze complex brain disorders such as schizophrenia from expression gene microarrays [80].

Sequencing technologies have undergone major improvements in recent decades to determine evolutionary changes in genetic, epigenetic mechanisms, and in the ‘MOLECULAR-CLASSIFICATION’, a topic that gained prominence as a cluster in the first period. An example of this can be found in a study published in 2010 which combined a global optimization algorithm called Dongguang Li (DGL) with cancer diagnostic methods based on gene selection and microarray analysis. It performed the molecular classification of colon cancers and leukemia and demonstrated the importance of machine learning, data mining, and good optimization algorithms for analyzing microarray data in the presence of subsets of thousands of genes [81].

The cluster ‘PROSTATE-CANCER’ in the second period (Figure 6, 2004–2012) presents a higher conceptual nexus to ‘MOLECULAR-CLASSIFICATION’ in the first sub-period and the same happens with clusters, such as ‘METASTASIS’, ‘BREAST-CANCER’, and ‘ALZHEIMER’, which appear more recently in the third sub-period. The significant increase in the incidence of prostate cancer in recent years results in the need for greater understanding of the disease in order to increase patient survival, since prostate cancer with metastasis was not well explored, despite having a survival rate much smaller compared to the early stages. In this sense, the understanding of age-specific survival of patients with prostate cancer in a hospital in using machine learning started to gain attention by academics and highlighted the importance of knowing survival after diagnosis for decision making and better genetic counseling [82]. In addition, the relationship between prostate cancer and Alzheimer’s disease is explained by the fact that the use of androgen deprivation therapy, used to treat prostate cancer, is associated with an increased risk of Alzheimer’s disease and dementia [81]. Therefore, the risks and benefits of long-term exposure to this therapy must be weighed. Finally, the relationship between prostate cancer and breast cancer in the thematic evolution can be explained due to the fact that studies are showing that men with a family history of breast cancer have a 21% higher risk of developing prostate cancer, including lethal disease [83].

The cluster ‘PHARMACOVIGILANCE’ appears in the second sub-period (Figure 6, 2004–2012) showing a strong co-occurrence with clusters of the third sub-period: ‘ADVERSE-DRUGS-REACTIONS’ and ‘ELECTRONIC-HEALTH-RECORDS’. In recent years, data-mining algorithms have stood out for their usefulness in detecting and screening patients with potential adverse drug reactions and, consequently, they have become a central component of pharmacovigilance, important for reducing the morbidity and mortality associated with the use of medications [48]. The importance of electronic medical records for pharmacovigilance is evident, which act as a health database and enable drug safety assessors to collect information. In addition, such medical records are also essential to optimize processes within health institutions, ensure more safety of patient data, integrate information, and facilitate the promotion of science and research in the health field [84]. These characteristics explain the large number of studies of ‘ELECTRONIC-HEALTH-RECORDS’ in the third sub-period and the growth of this theme in recent years, since the world has started to introduce electronic medical records, although currently there are few institutions that still use physical medical records.

The ‘DEPRESSION’ appears in the second sub-period (Figure 6, 2004–2012) and remains as a trend in the third sub-period with a significant increase in publications on the topic. It is known that this disease is numerous and is increasing worldwide, but that it still has many stigmas in its treatment and diagnosis. Globalization and the contemporary work environment [85] can be explanatory factors for the increase in the theme from the 2000s onwards and the COVID-19 pandemic certainly contributed to the large number of articles on mental health published in 2020. In this context, improving the detection of mental disorders is essential for global health, which can be enhanced by applying data mining to quantitative electroencephalogram signals to classify between depressed and healthy people and can act as an adjuvant clinical decision support to identify depression [69].

## 5. Conclusions

In this research, we have performed a BPNA to depict the strategic themes, the thematic network structure, and the thematic evolution structure of the data mining applied in healthcare. Our results highlighted several significant pieces of information that can be used by decision-makers to advance the field of data mining in healthcare systems. For instance, our results could be used by editors from scientific journals to enhance decision-making regarding special issues and manuscript review. From the same perspective, healthcare institutions could use this research in the recruiting process to better align the position needs to the candidate’s qualifications based on the expanded clusters. Furthermore, Table 2 presents a series of authors whose collaboration network may be used as a reference to identify emerging talents in a specific research field and might become persons of interest to greatly expand a healthcare institution’s research division. Additionally, Table 3 and Table 4 could also be used by researchers to enhance the alignment of their research intentions and partner institutions to, for instance, encourage the development of data mining applications in healthcare and advance the field’s knowledge.

The strategic diagram (Figure 4) depicted the most important themes in terms of centrality and density. Such results could be used by researchers to provide insights for a better comprehension of how diseases like ‘CANCER’, ‘DIABETES-MELLITUS’, ‘ALZHEIMER’S-DISEASE’, ‘BREAST-CANCER’, ‘DEPRESSION’, and ‘CORONARY-ARTERY-DISEASE’ have made use of the innovations in the data mining field. Interestingly, none of the clusters have highlighted studies related to infectious diseases, and, therefore, it is reasonable to suggest the exploration of data mining techniques in this domain, especially given the global impact that the coronavirus pandemic has had on the world.

The thematic network structure (Figure 5) demonstrates the co-occurrences among clusters and may be used to identify hidden patterns in the field of research to expand the knowledge and promote the development of scientific insights. Even though exhaustive research of the motor themes and their subthemes has been performed in this article, future research must be conducted in order to depict themes from the other quadrants (Q2, Q3, and Q4), especially emerging and declining themes, to bring to light relations between the rise and decay of themes that might be hidden inside the clusters.

The thematic evolution structure showed how the field is evolving over time and presented future trends of data mining in healthcare. It is reasonable to predict that clusters such as ‘NEURAL-NETWORKS’, ‘FEATURE-SELECTION’, ‘EHR’ will not decay in the near future due to their prevalence in the field and, most likely, due to the exponential increase in the amount of patient health that is being generated and stored daily in large data lakes. This unprecedented increase in data volume, which is often of dubious quality, leads to great challenges in the search for hidden information through data mining. Moreover, as a consequence of the ever-increasing data sensitivity, the cluster ‘SECURITY’, which is related to the confidentiality of the patient’s information, is likely to remain growing during the next years as government and institutions further develop structures, algorithms, and laws that aim to assure the data’s security. In this context, blockchain technologies specifically designed to ensure integrity and publicity of de-identified, similarly as it is done by the MIMIC-III (Medical Information Mart for Intensive Care III) [78], may be crucial to accelerate the advancement of the field by providing reliable information for health researchers across the world. Furthermore, future researches should be conducted in order to understand how these themes will behave and evolve during the next years, and interpret the cluster changes to properly assess the trends here presented. These results could also be used as teaching material for classes, as it provides strategic intelligence applications and the field’s historical data.

In terms of limitations, we used the WoS database since it has index journals with high JIF. Therefore, we suggest to analyze other databases, such as Scopus, PubMed, among others in future works. Besides, we used the SciMAT to perform the analysis and other bibliometric software, such as VOS viewer, Cite Space, Sci2tool, etc., could be used to explore different points of view. Such information will support this study and future works to advance the field of data mining in healthcare. 

## Figures and Tables

**Table 1:** Existing bibliometric analysis of data mining in healthcare in Web of Science (WoS).

Study	Coverage	Focus
[14]	2000–2017	Analysis of the evolution of emerging technologies (e.g., data mining, machine learning, among others) in cancer using CiteSpace software.
[15]	2009–2018	Exploration of data mining and machine learning in public health sector.
[16]	2011–2019	Investigation of medical data mining using VOSviewer and CiteSpace software.
This paper	1995–2020	A BPNA of data mining in healthcare: performance analysis, strategic themes, thematic evolution structure, trends and future opportunities using SciMAT software.

**Table 2:** Most Cited/Productive authors from 1995 to July 2020.

Author Citation	Citations	Author Productivity	Documents
Bate, Andrew C.	945	Li, Chien-Feng	36
Lindquist, Marie	943	Acharya, U. Rajendra	21
Edwards, E.R.	888	Chung, Kyungyong	21
Moore, Jason H.	711	Chen, Gang	19
Cook, Diane, J.	599	Lee, Sung-Wei	18
Eppig, Janan, T.	577	Moore, Jason H.	17
White, Bill, C.	541	Cano, Maria	17
Bellazi, Riccardo	527	Chang, I-Wei	16
Szarfman, A.	511	He, Hong-Lin	16
Lambin, Philippe	489	Moro, Pedro L.	16

**Table 3:** Journals that publish studies to data mining in healthcare.

Journal	Doc.	JIF
PLOS One	124	2.74
Expert Systems with Applications	105	5.89
Artificial Intelligence in Medicine	75	4.47
Journal of Biomedical Informatics	75	3.57
BMC Bioinformatics	66	2.13
Journal of Medical Systems	65	2.83
IEEE Access	65	3.74
Computer Methods and Programs in Biomedicine	59	3.63
International Journal of Advanced Computer Science and Applications	54	1.32
Journal of The American Medical Informatics Association	53	4.11

**Table 4:** Institutions and countries that publish studies to data mining in healthcare.

University	Documents	Country	Documents
Columbia University	62	United States	1973
U.S. FDA Registration	62	China	923
Harvard University	60	England	370
Stanford University	55	India	354
Chinese Academy of Sciences	53	Germany	312
Chi Mei Medical Center	47	Italy	297
University of Pennsylvania	45	Taiwan	294
Kaohsiung Medical University	44	Australia	282
University of Toronto	44	Canada	252
University of Pittsburgh	44	Netherlands	117

**Table 5:** Most relevant WoS subject categories and research fields.

WoS Subject Categories	Doc.
Computer Science Artificial Intelligence	768
Medical Informatics	744
Computer Science Information Systems	722
Computer Science Interdisciplinary Applications	603
Mathematical Computational Biology	505
Health Care Sciences Services	419
Pharmacology Pharmacy	370
Engineering Electrical Electronic	364
Computer Science Theory Methods	357
Biochemical Research Methods	304

## Data Availability

Not applicable.
